# The COBLL1 C allele is associated with lower serum insulin levels and lower insulin resistance in overweight and obese children

**DOI:** 10.1002/dmrr.2408

**Published:** 2013-07-05

**Authors:** Rosellina Margherita Mancina, Maria Antonella Burza, Cristina Maglio, Carlo Pirazzi, Federica Sentinelli, Michela Incani, Tiziana Montalcini, Arturo Pujia, Tiziana Congiu, Sandro Loche, Sabrina Pilia, Olov Wiklund, Jan Borén, Stefano Romeo, Marco Giorgio Baroni

**Affiliations:** 1 Institute of Medicine, Sahlgrenska Center for Cardiovascular and Metabolic Research, Department of Molecular and Clinical Medicine, Wallenberg Laboratory, University of GothenburgSweden; 2 Clinical Nutrition Unit, Department of Medical and Surgical Sciences, University Magna Graecia of CatanzaroItaly; 3 Endocrinology and Diabetes, Department of Medical Sciences, University of CagliariItaly; 4 Pediatric Endocrine Unit, Regional Hospital for MicrocitaemiaCagliari, Italy; 5 Endocrinology, Department of Experimental Medicine, Sapienza University of RomeItaly

**Keywords:** COBLL1, insulin resistance, children, genetics, obesity, rs7607980

## Abstract

**Background** Childhood obesity is a growing epidemic worldwide, and it is associated with metabolic complications, such as insulin resistance. Recently, a genetic variation (rs7607980) in the COBLL1 gene has been associated with lower insulin resistance in adults. The aim of the study was to investigate if the association between COBLL1 rs7607980 genetic variant and lower insulin resistance was present early in life.

**Methods** This sequence variant was genotyped in 878 overweight and obese children (mean age: 10 years) from Sardinia, Italy, from the outpatient clinic of the Pediatric Endocrine Unit, at the Regional Hospital for Microcitaemia in Cagliari. Insulin resistance was assessed by measurement of fasting circulating insulin levels before and after an oral glucose tolerance test and by HOMA-IR.

**Results** The COBLL1 rs7607980 C allele was associated with lower fasting insulin and HOMA-IR levels (*p* = 0.002 and *p* = 0.035, respectively) in overweight and obese children. Importantly, lower insulin levels were also observed 2 h after oral glucose tolerance test in C allele carriers (*p* = 0.009).

**Conclusions** The present study shows for the first time, the association between COBLL1 rs7607980 C allele, lower serum insulin levels and lower insulin resistance in overweight and obese children. Copyright © 2013 John Wiley & Sons, Ltd.

## Introduction

The prevalence of obesity in children is increasing rapidly worldwide. In the United States and in Europe, the prevalence of overweight/obese children is greater than 20% 1–2. Overweight/obesity prevalence in Italy mirrors the European proportion, with the highest prevalence of paediatric obesity reported in Southern Italy 3. Higher BMI is frequently associated with metabolic complications, including insulin resistance 4,5. Insulin resistance is characterized by impaired sensitivity of body tissues to the action of insulin, leading to high circulating insulin levels 7. This condition is a well-known risk factor for the development of type 2 diabetes 7. Genetic background plays a role in determining insulin resistance in both adults and children 8,9. A recent genome-wide association study on insulin resistance carried out in a large sample of adult individuals identified for the first time a common nonsynonymous variant (rs7607980) in the COBLL1 gene to be associated with lower insulin resistance 10. Importantly, an interaction between this sequence variant and BMI on insulin resistance was observed. The rs7607980 in COBLL1 gene identifies a thymine to cytosine substitution, which results in an asparagine to aspartic acid change at position 939 in the aminoacidic sequence, and the C allele was found associated with lower fasting insulin levels.

It is currently unknown whether this association is present only in adults or it also affects this metabolic trait early in life. Thus, the aim of this study was to investigate the effect of the COBLL1 rs7607980 genetic variant on insulin action in a cohort of children from Sardinia, in Southern Italy. To exploit the interaction between this genetic variant and BMI on insulin resistance, we examined only overweight and obese children.

## Materials and methods

### Subjects

The study cohort has been previously described 11. From the initial study cohort, the number of individuals increased from 535 to 878 for a 6-month extension of the enrolment period. Briefly, all the overweight/obese children, from the outpatient clinic of the Pediatric Endocrine Unit for excess body weight, at the Regional Hospital for Microcitaemia in Cagliari, Italy, were consecutively recruited (N = 878) from May 2007 to November 2008. Children with endocrine disorders or genetic syndromes, including syndromic obesity, were not included. None of the subjects were under medication. The study was approved from the Local Ethical Committee, and informed consent was obtained from children or their legal guardians.

### Anthropometric measurements

Body mass index standard deviation score was defined according to Italian growth charts in people aged 2–20 years 12. SDS-BMI >1 and >2 were used to define overweight and obesity, respectively. Pubertal development stages were determined in conformity with Tanner scale 13, and subjects were divided into two groups: prepubertal (Tanner’s stage I: boys with pubic hair and gonadal stage I, girls with pubic hair and breast stage I) and pubertal (Tanner’s stages II–V: boys with pubic hair and gonadal stage ≥II, girls with pubic hair and breast stage ≥II).

### Clinical and metabolic parameters

Fasting glucose, insulin, total cholesterol, LDL and HDL cholesterol and triglyceride levels were measured in all children. To evaluate insulin resistance, we calculated the HOMA-IR according to the formula: (fasting insulin μU/mL × fasting glucose mmol/L)/22.5 14. After an overnight fast, all subjects underwent an oral glucose tolerance test (OGTT) performed according to clinical recommendations for children 15. Briefly, 1.75 g of glucose/kg (up to a maximum of 75 g) was administered orally; blood samples were obtained before the OGTT (indicated as 0’) and 2 h after the OGTT (indicated as 120’) to measure plasma glucose and insulin. Type 2 diabetes was defined according to the American Diabetes Association criteria 16.

### Genotyping of COBLL1 rs7607980

According to manufacturer’s instructions, COBLL1 rs7607980 variant was genotyped by TaqMan® assay (Applied Biosystems, Foster City, CA, USA). Primers and probes (FAM and VIC labelled) were supplied directly by Applied Biosystems. The ABI Prism Sequence Detection System ABI 7900HT (Applied Biosystems) was used for post-PCR allelic discrimination by measuring allele-specific fluorescence. Data were extrapolated using the Sequence Detection Software (Applied Biosystems). Genotyping success rate was 100%.

### Statistical analyses

Continuous variables were described as means ± standard deviations or median and interquartile range. Categorical variables were shown as number and proportion. General linear model analysis under an additive model was used to assess the effect of the COBLL1 genotypes on continuous variables adjusting for age, gender, SDS-BMI and Tanner stage. Non-normally distributed variables were log-transformed before entering the statistical models. Genotype and allele as well as categorical variable distributions across the genotypes were compared using *χ*^2^ test. Statistical analyses were carried out using the IBM Statistical Package for Social Sciences (IBM SPSS, version 19.0, Inc. Chicago, IL, USA). Two-sided *p* values <0.05 were considered statistically significant.

## Results

A total of 878 Italian children from Sardinia were examined. The mean age of our study population was 10 ± 3 years, ranging from 2 to 19 years. All subjects were overweight or obese with a mean BMI of 27 ± 4 kg/m^2^ and a mean SDS-BMI of 1.9 ± 0.5. Children of male gender were 411 (47%), and the proportion of prepubertal individuals was 70%. The rs7607980 genotype and allele frequencies were in Hardy–Weinberg equilibrium (*p* = 0.999) with a minor allele frequency of 0.18. A total of 874 overweight/obese children were included in the analyses after the exclusion of four individuals with diagnosis of diabetes. Clinical characteristics of nondiabetic individuals stratified by COBLL1 genotypes are shown in Table 1. The C allele was associated with both lower fasting insulin levels (*p* = 0.002) and lower HOMA-IR (*p* = 0.035) after adjusting for age, gender, SDS-BMI and Tanner stage. Lower insulin levels in carriers of the C allele were also observed 2 h after ingestion of an oral glucose bolus (OGTT, see Research Design and Methods, Clinical and metabolic parameters for more details) (*p* = 0.009, Table 1). No other differences in indices of adiposity (i.e. BMI and SDS-BMI) or other metabolic characteristics across genotypes were observed (Table 1).

**Table 1:** Clinical characteristics stratified by COBLL1 (rs7607980) genotype in nondiabetic subjects

	COBLL1 rs7607980 genotype			
	TT	TC	CC	*p* value
N	587	259	28	—
Demographic				
Age	10 ± 3	11 ± 3	10 ± 3	0.599
Male gender, N (%)	265(45)	129(50)	16(57)	0.194
Prepubertal state, N (%)^a^	412(70)	179(69)	22(79)	0.616
Anthropometric				
BMI, kg/m^2^	27 ± 4	27 ± 4	27 ± 5	0.629
SDS-BMI^b^	2.0 ± 0.5	1.9 ± 0.5	1.8 ± 0.7	0.535
Glucose metabolism^c^				
Glucose 0’, mg/dL	88 ± 7	89 ± 7	89 ± 7	0.908
Glucose 120’, mg/dL	105 ± 17	104 ± 17	102 ± 13	0.421
Insulin 0’, μU/mL	13(8–20)	12(8–16)	14(9–28)	0.002
Insulin 120’, μU/mL	51(30–81)	42(30–69)	48(34–84)	0.009
HOMA-IR, U	3.1(1.8–4.5)	2.8(1.8–3.6)	3.3(1.8–5.9)	0.035
Lipid metabolism				
Total cholesterol, mg/dL	170 ± 32	164 ± 32	166 ± 29	0.074
HDL cholesterol, mg/dL	52 ± 12	52 ± 13	54 ± 10	0.685
LDL cholesterol, mg/dL	105 ± 29	100 ± 27	99 ± 23	0.062
Triglycerides, mg/dL	55(39–79)	53(37–74)	53(44–63)	0.604

Continuous variables are shown as means ± standard deviation or as median and interquartile range. Categorical variables are presented as number and proportion. Categorical variable distribution across the genotypes was compared by χ^2^ test. The *p* values for continuous variables were calculated using a general linear model analysis under an additive model after adjusting for age, gender, SDS-BMI and Tanner stage. Non-normally distributed variables were log-transformed before entering the model.

a Prepubertal state was defined as Tanner’s stage I: boys with pubic hair and gonadal stage I; girls with pubic hair and breast stage I.

b SDS-BMI was defined according to Italian growth charts in people aged 2–20 years.

c Glucose metabolism indices were measured after an overnight fast (0’) and 2 h (120’) after an oral glucose tolerance test (OGTT) performed according to clinical recommendations for children (1.75 g of glucose/kg, up to a maximum of 75 g, administered orally).

## Discussion

In the current report, we show for the first time an association between COBLL1 rs7607980 C allele, lower insulin levels and lower insulin resistance in overweight and obese children.

In a recent genome-wide association study, Manning *et al*. showed the association of the rs7607980 C allele with lower insulin resistance in adult Europeans. In this study an interaction between the genetic variant and BMI was observed 10. Thus, to exploit this interaction and to further elucidate the role of this genetic variant on glucose metabolism, we genotyped COBLL1 rs7607980 variant in overweight and obese children from Southern Italy.

We show that the association between the COBLL1 C allele and lower insulin resistance is present already in young individuals (mean cohort age: 10 years). Notably, the association of the C allele and lower insulin levels is also present 2 h after the ingestion of an oral bolus of glucose, confirming the findings on insulin levels observed in fasting condition.

Impaired insulin action is a major risk factor for type 2 diabetes development. Strategies aiming to reduce insulin resistance are known to lead to a decrease in type 2 diabetes incidence 17. Because the association between the COBLL1 C allele and lower insulin resistance is present early in life and thus it is likely to persist over time, it would be interesting to examine if the changes in insulin levels are also associated with a reduction of the long-term risk for type 2 diabetes. The identification of genetic variants involved in the susceptibility to insulin resistance may be a valuable tool to stratify the at-risk population, leading to tailored strategies for prevention and treatment of diabetes mellitus. In conclusion, we show for the first time the association between COBLL1 rs7607980 C allele, lower insulin levels and lower insulin resistance in overweight and obese children. Further studies are warranted to examine the effect of this genetic variant on type 2 diabetes susceptibility.

## Abbreviations

COBLL1: cordon-bleu protein-like 1
TT: individuals with two T alleles
TC: heterozygotes
CC: individuals with two C alleles
N: number
BMI: body mass index
SDS-BMI: body mass index standard deviation score
HOMA-IR: homeostastic model assessment of insulin resistance
HDL: high-density lipoprotein
LDL: low-density lipoprotein
